# Expression of the ether-a-gò-gò-related gene 1 channel during B and T lymphocyte development: role in BCR and TCR signaling

**DOI:** 10.3389/fimmu.2023.1111471

**Published:** 2023-09-08

**Authors:** Cesare Sala, Martina Staderini, Tiziano Lottini, Claudia Duranti, Gabriele Angelini, Gabriela Constantin, Annarosa Arcangeli

**Affiliations:** ^1^ Department of Experimental and Clinical Medicine, Section of Internal Medicine, University of Florence, Florence, Italy; ^2^ Department of Medicine, Section of General Pathology, University of Verona, Verona, Italy

**Keywords:** Ca 2+ signalling1, BCR and TCR signalling2, ERG1 K + channel3, B and T lymphocytes development4, SOCE Ca 2+ overload5, Kv1.3 channel6, ERK phosphorylation

## Abstract

The functional relevance of K^+^ and Ca^2+^ ion channels in the “Store Operated Calcium Entry” (SOCE) during B and T lymphocyte activation is well proven. However, their role in the process of T- and B- cell development and selection is still poorly defined. In this scenario, our aim was to characterize the expression of the ether à-go-go-related gene 1 (ERG1) and K_V_1.3 K^+^ channels during the early stages of mouse lymphopoiesis and analyze how they affect Ca^2+^signaling, or other signaling pathways, known to mediate selection and differentiation processes of lymphoid clones. We provide here evidence that the mouse (m)ERG1 is expressed in primary lymphoid organs, bone marrow (BM), and thymus of C57BL/6 and SV129 mice. This expression is particularly evident in the BM during the developmental stages of B cells, before the positive selection (large and small PreB). mERG1 is also expressed in all thymic subsets of both strains, when lymphocyte positive and negative selection occurs. Partially overlapping results were obtained for K_V_1.3 expression. mERG1 and KV1.3 were expressed at significantly higher levels in B-cell precursors of mice developing an experimental autoimmune encephalomyelitis (EAE). The pharmacological blockage of ERG1 channels with E4031 produced a significant reduction in intracellular Ca^2+^ after lymphocyte stimulation in the CD4^+^ and double-positive T-cell precursors’ subsets. This suggests that ERG1 might contribute to maintaining the electrochemical gradient responsible for driving Ca^2+^ entry, during T-cell receptor signaling which sustains lymphocyte selection checkpoints. Such role mirrors that performed by the shaker-type K_V_1.3 potassium channel during the activation process of mature lymphocytes. No effects on Ca^2+^ signaling were observed either in B-cell precursors after blocking K_V_1.3 with PSORA-4. In the BM, the pharmacological blockage of ERG1 channels produced an increase in ERK phosphorylation, suggesting an effect of ERG1 in regulating B-lymphocyte precursor clones’ proliferation and checkpoint escape. Overall, our results suggest a novel physiological function of ERG1 in the processes of differentiation and selection of lymphoid precursors, paving the way to further studies aimed at defining the expression and role of ERG1 channels in immune-based pathologies in addition to that during lymphocyte neoplastic transformation.

## Introduction

B- and T-cell developments are strictly controlled processes that ensure the selection of mature lymphocytes expressing a correctly rearranged and not autoreactive B- or T-cell receptor (BCR or TCR). B-cell precursors in the bone marrow (BM) are firstly selected on the basis of the expression of a functional pre-BCR (1–5), passing from large pre-B to small pre-B stages (Hardy fractions C′ and D (6)). Finally, those precursors that correctly express a functional IgM receptor on their surface (positive selection) and that do not recognize self-antigens (negative selection) are selected and develop into mature B cells. Immature lymphocytes that fail to rearrange the BCR locus die from apoptosis (1, 7).

Similarly, thymocytes at the CD4 CD8 double-negative (DN3) stage that correctly rearranged the β chain of TCR (β-selection) (8, 9), receive pro-survival signals and differentiate into double-positive (DP) T lymphocytes. T-lymphocyte precursors at this stage are selected depending on the affinity of their rearranged TCRαβ receptors for MHC-self peptide (positive–negative selection) and undergo lineage commission, differentiating in CD4 and CD8 single positive (hereafter abbreviated as CD4SP and CD8SP) (8, 10).

Such selection checkpoints are mediated by BCR and TCR affinity for their ligands, which is transduced into different extents of Ca^2+^ influx, similarly to mature lymphocyte activation, mediating critical lymphocyte cellular responses and effector functions including cytokine production, selection, differentiation, and cytotoxicity (11). Such signaling is regulated by a fine-tuned network of ion channels and plasma membrane transporters that modulate K^+^ and Ca^2+^ currents (12). TCR and BCR antigen binding promotes an influx of Ca^2+^ (up to 1 μM) through CRAC channels, which depends on the negative membrane potential (Vm). The latter is mostly maintained by K^+^ and, although to a letter extent, by TRPM4 channels (11, 13, 14).

The most abundant and best-characterized K^+^ channels in human and murine T cells are the voltage-activated K^+^ channel K_V_1.3 and the Ca^2+^-activated K^+^ channel KCa_3.1_. K_V_1.3 is preferentially upregulated over KCa_3.1_, in response to antigen- or mitogen-specific activation to facilitate K^+^ efflux, which hyperpolarizes Vm and in turn controls Ca^2+^ entry (15–18). The deregulation of these K^+^ channels is implicated in abnormal lymphocyte signaling, affecting selection and regulatory cell activation (19–21), which are emerging as important drug targets affecting Ca^2+^ signaling (22–25).

K_V_1.3 and KCa_3.1_ expression levels vary in the different lymphoid subsets and state of activation (15, 26–28). However, few studies have been carried out on the expression and role of these K^+^ channels in modulating BCR- and TCR-mediated Ca^2+^ signaling during lymphocyte development checkpoints. Only one study showed a role for a different ion channel, a voltage-gated Na^+^ channel (VGSC), during the positive selection of CD4SP T cells, providing a mechanism whereby a weak signal of positive selection can induce a significant influx of Ca^2+^ that is required for CD4SP T-cell development. Consistently, its blockage inhibits CD4SP differentiation (29).

The K^+^ channels mentioned above have also been implicated in autoimmune diseases. For example, K_V_1.3 confers a survival advantage on proliferating autoreactive clones (15, 30, 31), where abnormalities in the pERK signaling pathways have been reported (32). Overall, K_V_1.3 is emerging as an immunomodulator target in autoimmune diseases (16, 33).

Finally, K^+^ channels are often aberrantly expressed in acute and chronic lymphocyte leukemias (ALL and CLL, respectively) as well as in lymphomas (34–38). Among them, the human ether à-go-go-related gene 1 (hERG1, encoded by Kv11.1) is particularly relevant. In B-ALL, hERG1 sustains the occurrence of chemoresistance (39). In acute myeloid leukemias (AML), hERG1 regulates cell motility and trans-endothelial migration, and its expression correlates with a worse prognosis (36). Interestingly, leukemias often preferentially express the hERG1b isoform (40, 41), which lacks the first five exons of the gene, being substituted by a small 1b exon, whereas the remaining sequence of the channel, including the pore region, remains unvaried (41).

Interestingly, both autoimmune diseases and leukemias are often associated with the aberrant selection of lymphocyte precursors during lymphopoiesis, which occurs when abnormal mutations accumulate in the DNA of developing precursors of lymphocytes within the bone marrow or lymphoid organs (11, 18, 42–44).

During development, the ERG1 transcript was found to be expressed in the embryonic heart and CNS and later in ganglia, retina, skeletal muscle, and other tissues, despite its function in developing tissues remaining largely unknown. It is reasonable to assume that the morphogenetic activities of ERG1 are involved in regulating cell proliferation/differentiation and migration (revised in (45)).

In lymphoid organs, ERG1 was reported to be expressed at a significant level in the thymus of rat (46), mouse (47), and human (The Human Protein Atlas https://www.proteinatlas.org/ENSG00000100346-CACNA1I/immune+cell).

Overall, a potential role of ERG1 during B- and T-lymphocyte development and autoreactive or in the expansion of neoplastic clones is emerging. This forced us to investigate the ERG1 expression and role in Ca^2+^ signaling, or other signaling pathways, during the lymphopoietic process in mice.

## Materials and methods

### Purification of murine lymphoid primary cells

The 10 C57BL/6 WT (Envigo), three induced EAE C57BL/6 provided by Prof. Gabriela Constantin, University of Verona), and three SV129 WT (Envigo) male and female mice sacrificed at the age of 3 months (except when specified), to avoid the process of thymic regression. Animals were sacrificed by CO_2_ inhalation, and all procedures were carried out in accordance with the European directive 86/609/EEC. This project has been approved by the Italian Ministry of Health with the authorization number 721/2019. Murine procedures were performed at the animal house (CeSAL), in the LIGeMA laboratory, at the University of Florence, Italy.

The different lymphoid organs were explanted and cells passed through a 70-μm filter to obtain a single-cell suspension and then treated with Red Cells Lysis Buffer (8.3 g NH4Cl, 1.0 g KHCO_3_, and 1.8 ml of 5% EDTA in 1 l distilled H_2_0).

### Western blot

WB analysis was performed as in (48) using 1:1,000 anti-phospho-p44/42 MAPK (Erk1/2) (Thr202/Tyr204) antibody (Polyclonal, Cell Signaling) and 1:200 anti-ERK 1 (C-16): sc-93 antibody (Polyclonal, Santa Cruz).

### Densitometric analysis

WB densitometric analysis was carried out using ImageJ software (ImageJ v.1.38, U.S. National Institutes of Health). We have evaluated three different scans after subtracting the background.

For pERK quantification, the signal for the protein was divided by the signal of the total protein ERK. It is worth noting that the ERK protein appears as a doublet and both bands, visible in each line of the blot, were considered for densitometric quantification. The resulting value is indicated as “pERK/ERK” throughout the manuscript and in the figures. At least three independent experiments were performed. Statistical comparisons were performed with OriginPro 2015 and SAS 9.2 (SAS Institute) software, following the application of the test reported in the paragraph labelled as “Statistics” in the Materials and Methods section.

Flow cytometry (FC) analysis was performed on an BD LSRII cytometer using the antibody staining panel reported in **Table 1**. The anti-ERG1 antibody was developed and patented by us. It was raised against and recognizes the human isoform of ERG1 (hERG1) (49), but it was proven to recognize the murine isoform as well (48), since human-murine homology for this gene is >96% (see **Supplementary Figure S1**). The antibody recognizes an extracellular epitope of the hERG1 channel, and hence, when used without permeabilization, it only recognizes the channel expressed on the plasma membrane (50). The gating strategy we followed is reported in **Supplementary Figure 2**.

**Table 1:** B- and T-precursor staining panel for cytometric analysis.

T-cell staining panel		Clone	Dilution	Source
CD4	AF647	RM4-5	1/800	BioLegend
CD8	APC-Cy7	53-6.7	1/300	BioLegend
CD44	PE	IM7	1/300	BD
CD25	PE-Cy7	PC 61.5	1/800	eBioscience
Tcrβ	PE-Cy5	H57-597	1/600	BioLegend
ERG1	AF488	A7	1/100	MCK Therapeutics
Kv1.3	AF488	Polyclonal	1/100	Alomone Labs
B-cell staining panel		Clone	Dilution	Source
IgM	AF647	RMM-1	1/600	BioLegend
CD43	PE-Cy7	S11	1/600	BioLegend
B220	APC-Cy7	RA3-6B2	1/600	BioLegend
ERG1	AF488	A7	1/100	MCK Therapeutics

### B-cell activation

B cells were stimulated with anti IgM mAb (RMM-1 BioLegend, 40 μg/ml) and anti-CD19 (MB19-1 SouthernBiotech, 20 μg/ml) immediately before the analysis.

### T-cell activation

T cells were incubated with anti-CD3 mAb (145-2C11 eBioscience, 20 μg/ml) for 30′ on ice, followed by washing and cross-linking with rabbit-anti-hamster IgG (Jackson ImmunoResearch, 75 μg/ml) immediately before the analysis.

### Intracellular Ca^2+^ measurement

Primary lymphocytes were stained with the Fluo-4 Ca^2+^ probe (Invitrogen, 2 µM) together with the antibodies reported in **Table 1** (except anti-ERG1) for 1 h at room temperature in the dark. Following PBS washing, lymphocytes were resuspended in RPMI containing 10% heat-inactivated bovine calf serum (HyClone) and supplemented with 2mM L-glutamine, at 37°C for 30′ before cytometer analysis. The Fluo-4 MFI was calculated for an interval of 30″ before (−) and after (+) the stimulation in the presence or in the absence of E4031 (Alomone Laboratories, Jerusalem, Israel, dissolved in water at [5 mM]), which was pre-incubated for 1 h at a concentration of 30 µM to avoid serum-related drug degradation that might mask the effect (51).

### Statistics

For the statistical analysis, paired t-tests were used. Data are presented as mean ± SD. Kolmogorov–Smirnov test was used to determine whether the data distribution was normal. Prism 8.4.3 software (GraphPad Software, San Diego, CA, USA) was used to conduct statistical analysis. All statistical tests were two-tailed with a significance level of 0.05.

## Results

### mERG1 protein expression in mouse hematopoietic tissues

**Figure 1:**
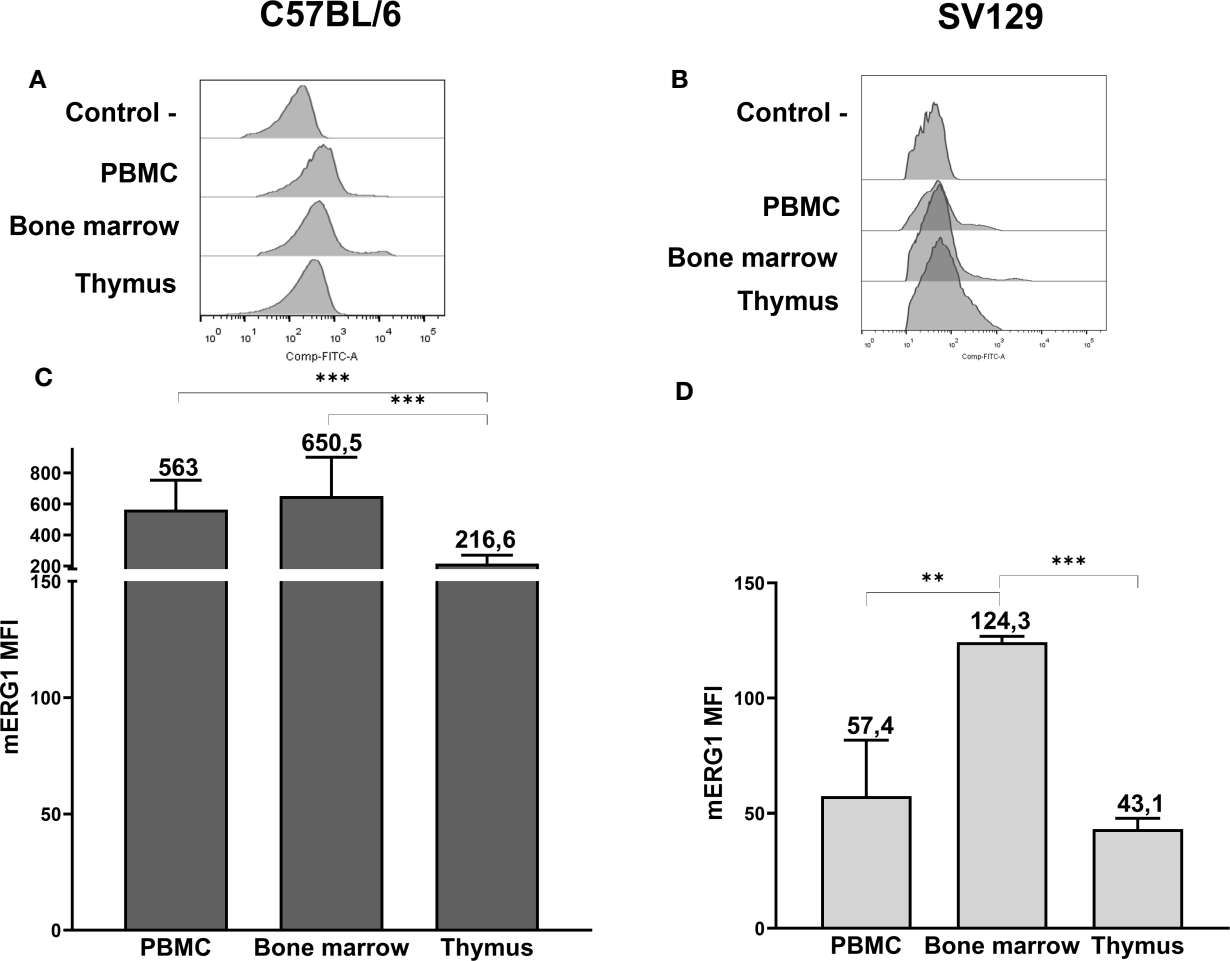
ERG1 expression in hemopoietic organs of C57BL/6 and SV129 mice. Representative histograms showing anti-ERG1 (AF488) fluorescence analyzed by flow cytometry of cells purified from different lymphoid organs of C57BL/6 **(A)** and of SV129 representative animals **(B)**. Control—histograms show the fluorescence of ERG1 or Kv1.3 unstained control. Bar graphs showing the mean fluorescence intensity (MFI) of cells purified from different lymphoid organs of C57BL/6 (n = 10) **(C)** and of SV129 (n = 3) strain **(D)**. Error bars indicate the standard deviation among mice of both sexes, 3 months of age. Statistical analysis was performed by two-tailed T-test (0.001 < p ≤ 0.01 **, p ≤ 0.001 ***).

We first characterized the expression of the murine (m)ERG1 in the two primary lymphoid organs, bone marrow (BM) and thymus, as well as in peripheral blood mononuclear cells (PBMC). Two different mouse strains, C57BL/6 and SV129, were used. In fact, it is known that the whole hematopoietic system may vary, in mice, depending on their strain (52). In addition, our choice was dictated from the different applications that our results could have for studies related to different pathologies (either immune-mediated or oncologic), which can be modeled in different mouse strains. mERG1 protein expression levels were determined by flow cytometry (FC), using a monoclonal antibody specific for an extracellular epitope of the human ERG1 protein, which shares a 96% homology with the murine ERG1 sequence (**Supplementary Figure S1**, **Figures 1A, B**). Single cells were purified from lymphoid organs and analyzed after gating on the lymphocyte population and after doublet exclusion (**Supplementary Figure 2**).

In the C57BL/6 strain, mERG1 turned out to be expressed in all the lymphoid organs we analyzed, with a significantly higher expression in the PBMC and BM compared with the thymus (**Figures 1A, C**). In SV129 mice, mERG1 was expressed at a slightly lower extent compared with C57BL/6 mice. Furthermore, a significantly higher mERG1 expression was detected in the BM, compared with both the PBMC and the thymus (**Figures 1B, D**).

### mERG1 expression in B- and T-lymphocyte subpopulations within primary lymphoid organs

**Figure 2:**
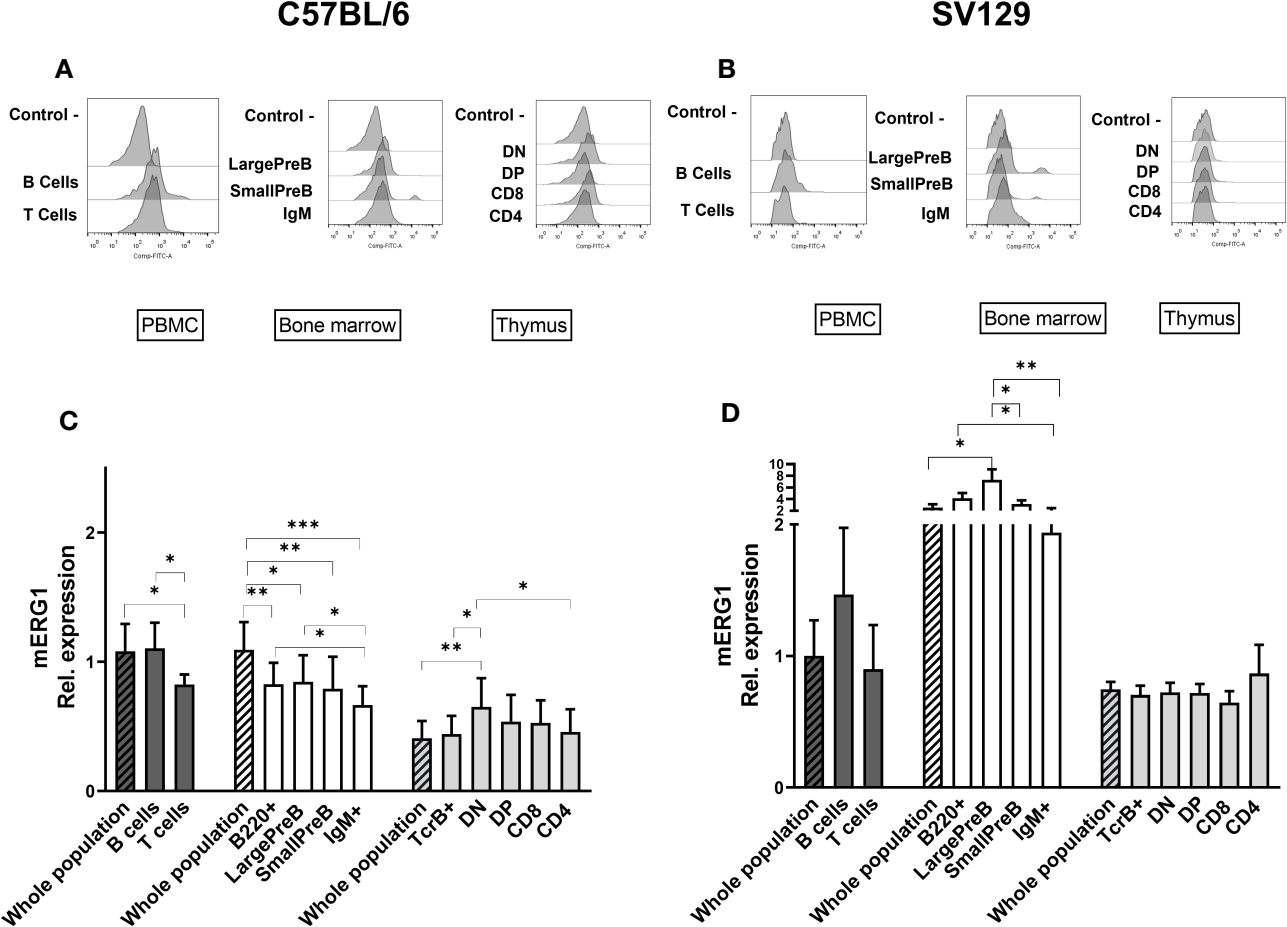
ERG1 expression in different lymphoid populations. Representative histograms showing anti-ERG1 (AF488) fluorescence analyzed by flow cytometry in the different lymphoid precursors’ subpopulations purified from different lymphoid organs of C57BL/6 **(A)** and of SV129 strain **(B)**. Control—histograms show the fluorescence of ERG1 or Kv1.3 unstained control. Bar graphs representing ERG1 expression levels in different lymphoid organs (bars marked with a line pattern) and subsets (bars with no pattern, gated as described in **Supplementary Figure 2**) of C57BL/6 (n = 10 **(C)** and SV129 (n = 3 **(D)** animals of 3 months of both sexes, normalized on the ERG1 MFI of the PBMC. Histograms represent the mean the fluorescence analyzed in the different animals, and error bars indicate the standard deviation. Statistical analysis was performed by two-tailed T-test (0.01 < p ≤ 0.05 *, 0.001 < p ≤ 0.01 **, p ≤ 0.001 ***).

Data provided in **Figure 1** prompted us to further analyze mERG1 expression in the different subpopulations of both B lymphocyte precursors, in the BM, and T lymphocyte precursors in the thymus. We applied the same FC approach used for **Figure 1**, performing a deeper analysis, based on the expression of specific surface markers, to distinguish the different developmental stages of lymphocyte precursors in the BM and thymus of both C57BL/6 and SV129 mouse strains. FC analysis was also applied to B- and T-cell populations from the PBMC. Single cells were purified from lymphoid organs of 10 C57BL/6 mice and three SV129 mice of both sexes, 3 months old, and distinguished into various subpopulations. In particular, B cells in the BM were distinguished into their main stages of maturation: B-cell precursors that have not yet expressed IgM (B220^+^ IgM−), which were further subdivided into the large pre-B (Hardy’s fraction C′) and small pre-B cell (fraction D) subsets, on the basis of their dimension and of the expression of the CD43 marker, and immature B cells that express IgM (B220 ^+^ IgM ^+^, fraction F) (**Supplementary Figure 2**). Similarly, thymocytes were first gated on TCRβ^+^ (intermediate and high expression levels) and then further subdivided into double-negative (DN), double-positive (DP), and CD8 and CD4 SP subsets, according to the expression of the CD4 and CD8 coreceptor markers (**Supplementary Figure 2**). In the whole set of different lymphocyte populations, the expression of the mERG1 was determined by FC as above. Since the antibody used for ERG1 detection recognizes an extracellular epitope of the channel protein, if used without permeabilization, it only detects ERG1 proteins expressed on the plasma membrane (50). Representative histograms relative to all the populations analyzed in C57BL/6 and SV129 mice are shown in **Figures 2A and B**, respectively. Bar graphs showing the AF488 mean fluorescence intensity (MFI) values relative to mERG1 expression in the different lymphocyte subsets from the PBMC, BM, and thymus of C57BL/6 and SV129 mice are shown in **Figures 2C, D**, respectively. Data are shown as mean ± SD, and mERG1 expression levels in the single-cell populations were normalized on the level of the protein in the whole PBMC population (shaded bars on the left of panels C and D).

In C57BL/6 mice, mERG1 was expressed at higher levels in the B cells (B220+) from PBMC compared with T cells (TCRβ+) (**Figures 2A, C**). In the BM, mERG1 was expressed in all the subsets, with a decrease in the last phase of development (IgM+). Similarly, in the SV129 strain, mERG1 was expressed at higher levels in the B cells both from the PBMC and from the BM compared with T cells. mERG1 expression in the different B-cell population of the BM is apparently higher in SV129 mice compared with C57BL/6 (compare **Figures 2C, D**). An increase in the expression in the large PreB population and a decrease in the last phase of development (IgM+) were detected in the BM of SV129 mice (**Figure 2D**). Among TCRβ^+^ thymocytes, in C57BL/6 mice, mERG1 was expressed at similar levels in all the thymic subsets, except in the DN population where its expression increased (**Figures 2A, C**). In SV129 mice, no significant variations in the expression of mERG1 emerged in the different TCRβ^+^ subsets, where only a slight increase in the CD4+ population was observed (**Figure 2D**). Also in this case, the mERG1 expression was normalized to the MFI of the whole PBMC population (**Figure 2D**, dark-gray bars).

Overall, data shown in **Figure 2** indicate significantly different expression levels between the two murine strains analyzed. In particular, both strains express mERG1 at higher levels in the first phases of B-cell development (large PreB and small PreB), but SV129 mice show a much higher mERG1 expression in B cells. In the thymus, mERG1 is more expressed in the early phases of T lymphocyte development (DN cells). This only occurs in mice of the C57BL/6 strain, whereas no difference in the different TCRβ^+^ thymocyte populations emerged in the SV129 strain.

### Kv1.3 protein expression in mouse hematopoietic tissues

**Figure 3:**
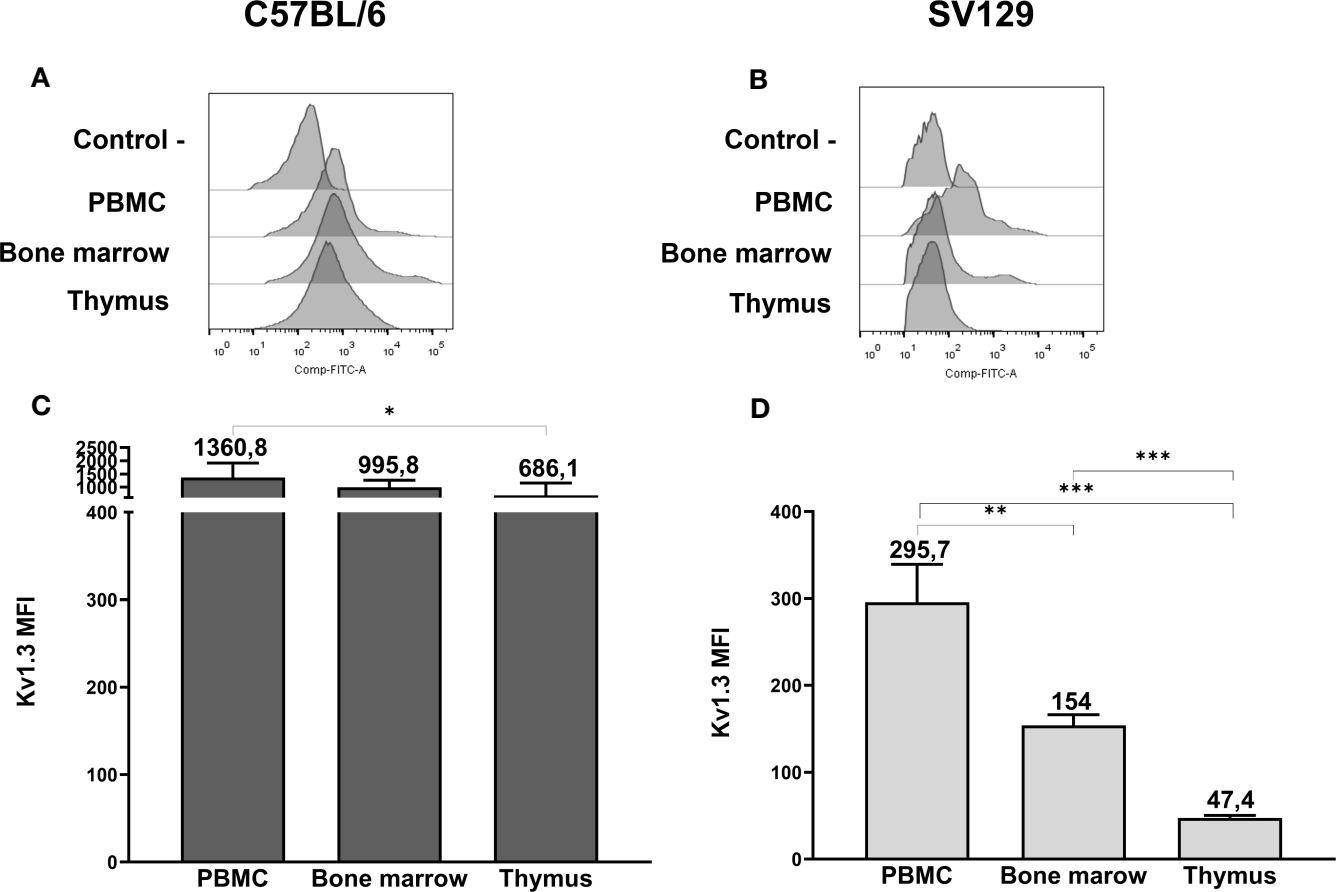
Kv1.3 expression in hemopoietic organs of SV129 mice. Representative histograms showing anti-Kv1.3 (AF488) fluorescence analyzed by flow cytometry of cells purified from different lymphoid organs of C57BL/6 **(A)** and of SV129 representative animals **(B)**. Control—histograms show the fluorescence of ERG1 or Kv1.3 unstained control. Bar graphs showing the mean fluorescence intensity (MFI) of cells purified from different lymphoid organs of C57BL/6 (n = 10) **(C)** and of SV129 (n = 3) strain **(D)**. Error bars indicate the standard deviation among mice of both sexes, 3 months aged. Statistical analysis was performed by two-tailed T-test (0.01 < p ≤ 0.05 *, 0.001 < p ≤ 0.01 **, p ≤ 0.001 ***).

**Figure 4:**
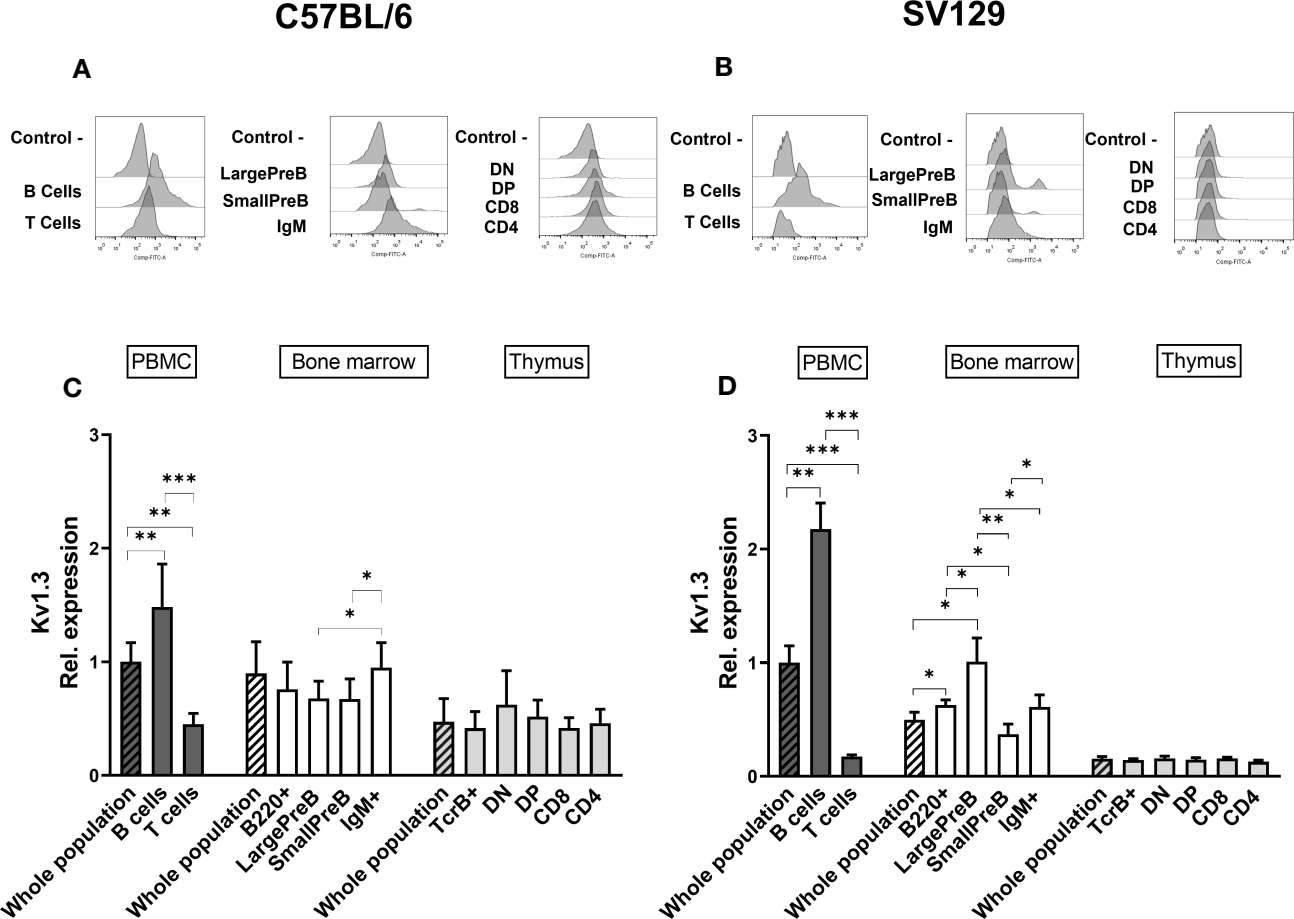
Kv1.3 expression in different lymphoid populations. Representative histograms showing anti-Kv1.3 (AF488) fluorescence analyzed by flow cytometry in the different lymphoid precursors’ subpopulations purified from different lymphoid organs of C57BL/6 **(A)** and of SV129 strain **(B)**. Control—histograms show the fluorescence of ERG1 or Kv1.3 unstained control. Bar graphs representing Kv1.3 expression levels in different lymphoid organs (bars marked with a line pattern) and subsets (bars with no pattern, gated as described in **Supplementary Figure 1**) of C57BL/6 (n = 10 **(C)** and SV129 (n = 3 **(D)** animals of 3 months of both sexes, normalized on the Kv1.3-AF488 MFI of the PBMC. Histograms represent the mean the fluorescence analyzed in the different animals, and error bars indicate the standard deviation. Statistical analysis was performed by two-tailed T-test (0.01 < p ≤ 0.05 *, 0.001 < p ≤ 0.01 **, p ≤ 0.001 ***).

To strengthen our data on the expression of mERG1 during lymphocyte development, we investigated the expression of the voltage-dependent K^+^ channel K_V_1.3, whose expression and role during mature lymphocyte activation is well established (15–18). In C57BL/6 mice, K_V_1.3 turned out to be expressed at high levels in PBMC, BM, and thymus, with a statistically different higher expression in PBMC compared with the thymus (**Figures 3A, C**). In SV129 mice, good expression levels of the channel were observed, with significantly lower levels in the BM and even more in the thymus, compared with peripheral blood (**Figures 3B, D**). In the single subpopulations of both C57BL/6 and SV129 strains, K_V_1.3 turned out to be expressed at higher levels in the B cells both in the PBMC and in the BM (**Figures 4A-D**). In the BM, K_V_1.3 expression showed an opposite trend to that observed for mERG1, being expressed at higher levels during the latest phase of B development (IgM^+^) in C57BL/6 mice—during the earliest phases (large PreB) in SV129 mice (**Figures 4C, D**). Similar to that observed for mERG1, K_V_1.3 expression was lower in the thymus than in the other lymphoid organs and no differences among the subpopulation were detected in both strains (**Figures 4C, D**).

We also assessed whether either mERG1 or K_V_1.3 expression levels varied with age. To this purpose, we analyzed the expression levels of the two channels in organs from older (7 and 14 months) SV129 animals. No differences in both the overall expression levels and the different expressions among the subsets were observed (**Supplementary Figure 3**).

### mERG1 and Kv1.3 expression in an induced experimental autoimmune encephalomyelitis murine model

**Figure 5:**
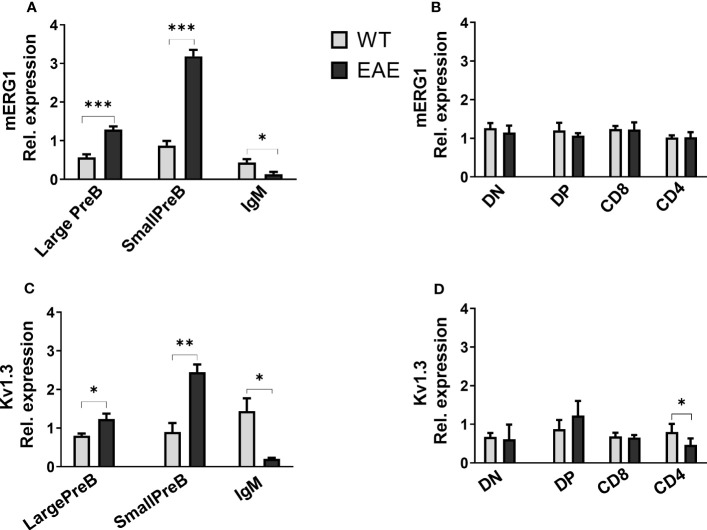
ERG1 and Kv1.3 expression in different lymphoid populations of the experimental autoimmune encephalomyelitis (EAE) disease murine model Histograms representing ERG1 and Kv1.3 expression levels in the BM **(A, C)** and in the thymus **(B, D)** of C57BL/6 (n = 10) and C57BL/6 EAE sacrificed at the peak of the disease, 15 days after the EAE induction (n = 3). Histograms represent the mean of the fluorescence analyzed in the different animals, normalized on the expression in PBMC; error bars indicate the standard deviation. Statistical analysis was performed by two-tailed T-test (0.01 < p ≤ 0.05 *, 0.001 < p ≤ 0.01 **, p ≤ 0.001 ***).

With the aim to get some insights on the role of mERG1 expression during lymphocyte development, we evaluated its expression in a murine model of induced EAE. Experiments were carried out in 3-month-old C57BL/6 mice, and the data relative to the EAE score of each animal are reported in **Supplementary Table 1**. mERG1 expression was higher during B lymphocyte development of EAE mice, compared with the untreated C57BL/6 animals, particularly in the large and small PreB subsets. On the contrary, its expression was lower in the IgM^+^ more mature subset (**Figure 5A**). No differences in the expression levels of the channel were observed in developing TCRβ^+^ thymocytes in the thymus (**Figure 5B**). A similar expression profile in the B populations emerged for K_V_1.3, which was more expressed in the large and small PreB subsets and reduced in the IgM^+^ population (**Figure 5C**). A slight reduction of K_V_1.3 expression in the CD4^+^ subset in the thymus emerged (**Figure 5D**).

### Role of mERG1 in Ca^2+^ signaling in B- and T-cell precursors

Data gathered so far, and in particular the rough correspondence in the expression profile of mERG1 and of the most characterized K_V_1.3 channel in both B- and T-cell precursors, and their alterations during EAE induction, prompted us to further deepen the role of mERG1 in Ca^2+^ signaling mechanisms which are associated with lymphocyte selection checkpoints during lymphoid development. These experiments were carried out only in mice belonging to the C57BL/6 strain, being the strain of choice of the EAE model.

**Figure 6:**
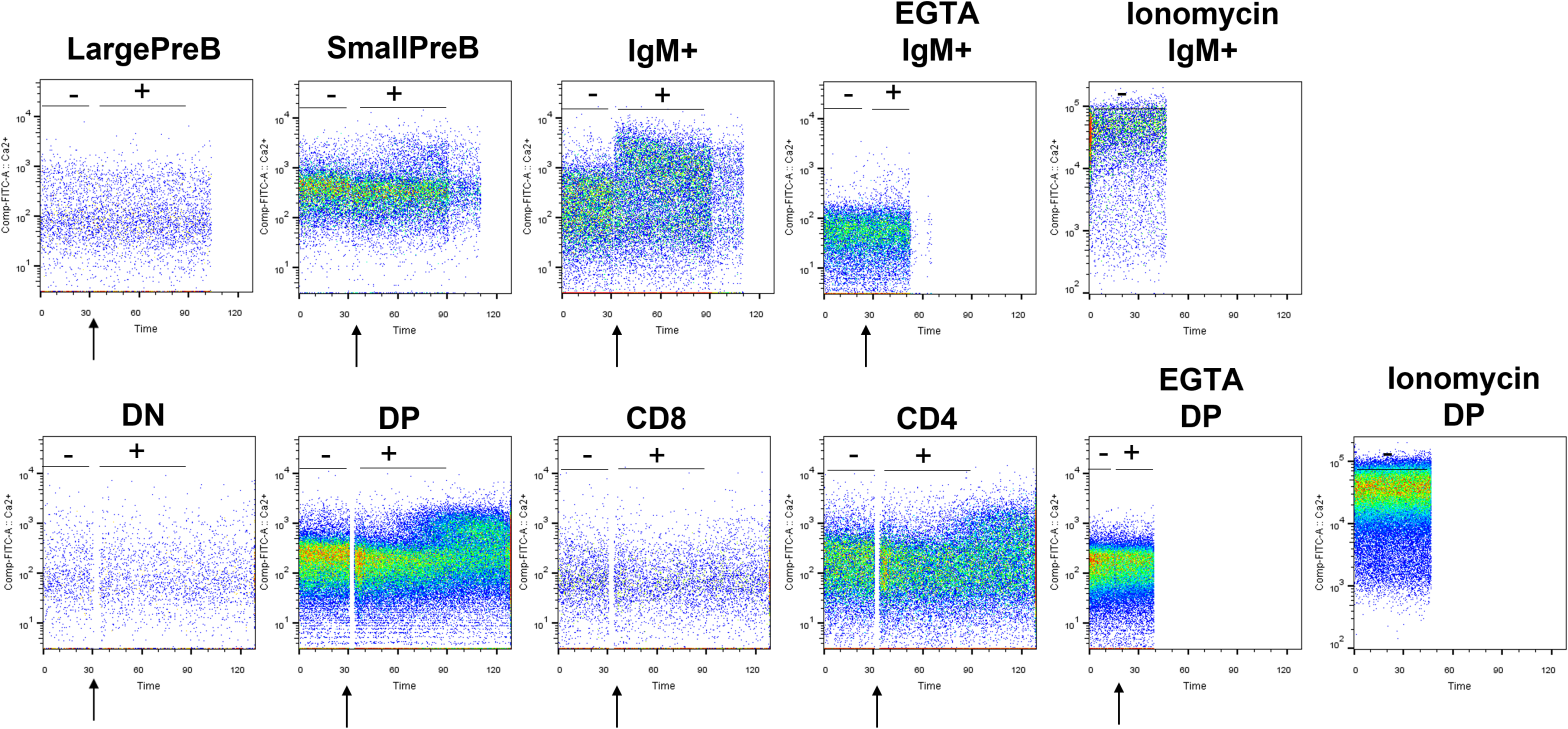
Representative plots showing the variation in Ca^2+^ level before and after BCR and TCR stimulation. Plots showing the Fluo-4 fluorescence in lymphocyte precursors purified from the BM and thymus of a 3-month male C57BL/6 mouse. After 1 min of acquisition, lymphocyte’s precursors have been stimulated with a mix of anti-IgM and anti-CD19 for B cells and with anti-CD3 pre-binding followed by cross-linking with goat anti-hamster IgG for T cells to appreciate the Ca^2+^ entry wave. As control, part of the B and T lymphocytes were treated with ionomycin and EGTA. The moment of the stimulation is indicated by the arrow. Tubes have been immediately reinserted in the cytometer to appreciate the SOCE. The Fluo-4 MFI was calculated before (−) and after (+) the stimulation.

**Figure 7:**
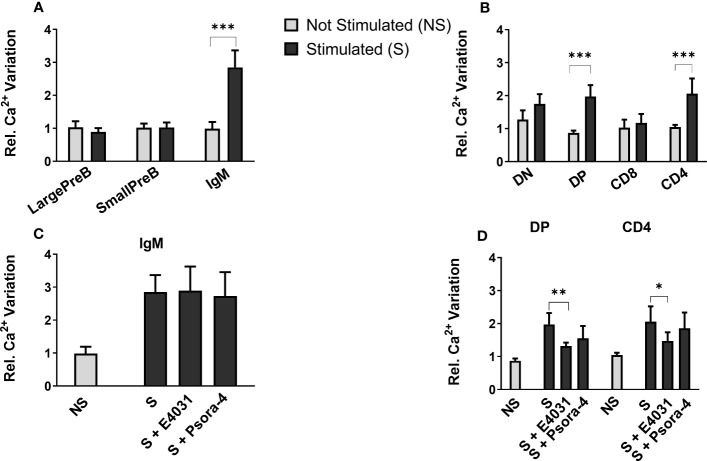
Effect of E4031 and Psora-4 on Ca^2+^ basal level and SOCE following BCR and TCR stimulation in the different lymphocytes’ precursor subsets. The Fluo-4 MFI in the different lymphocyte precursors from 7- and 3-month-old C57BL/6 mice of both sexes was calculated over a period of 1 min before (−) and 1.5 min after the stimulation (+). Bars represent the mean Fluo-4 MFI for each subset in the different experimental conditions, normalized for the untreated and unstimulated (unt, −) B220^+^ **(A)** or TCRβ^+^ population **(B)**. The effect of E4031 and Psora-4 treatment on the population that responded to the stimulation is shown in **(C, D)** Statistical analysis was performed by two-tailed T-test (0.01 < p ≤ 0.05 *, 0.001 < p ≤ 0.01 **, p ≤ 0.001 ***).

We hence determined intracellular Ca^2+^ variations, measuring the fluorescence emitted by Fluo-4, a dynamic single-wavelength fluorescent Ca^2+^ indicator, following the stimulation of the BCR and TCR in lymphocyte precursors, in the presence or absence of the specific ERG1 open channel blocker, E4031 (51). In these experiments, we focused only on the analysis of C57BL/6 mice. In the BM, it is possible to appreciate the increase in the fluorescence of the Fluo-4 probe in the previously identified B lymphocyte precursors, before (−) and after (+) the stimulation of the BCR with a cocktail of anti-IgM and anti-CD19 antibodies (the stimulation is indicated by the arrow in **Figure 6**). We chose this stimulation protocol, being the most physiological one. The Fluo-4 MFI was calculated for an interval of 1 min before (−) and 1.5 min after (+) stimulation (**Figure 6**), in the presence or in the absence of E4031 (**Figure 7**). Upon stimulation (+), an increase in the intracellular Ca^2+^ level was detectable only in the IgM^+^ population (**Figure 7A**), where a functional BCR is expressed, and hence might respond to the anti-IgM and CD19 cocktail. When treating the cells with E4031 [30 μM] for 2 h before the stimulation, no effects on intracellular Ca^2+^ influx were observed in the IgM^+^ subpopulation, which responded to the stimulation we applied (**Figure 7C**). The fluorescence emitted by the Fluo-4 Ca^2+^ indicator was also measured in thymocytes, in each T-cell precursors’ subset, before and after the stimulation of the TCR by anti-CD3 binding followed by cross-linking with a goat anti-hamster IgG (**Figure 6**). Upon stimulation (+), CD4SP and DP populations exhibited an increase in the Fluo-4 MFI. Notably, these thymic subsets express a functional TCR and hence might indeed respond to the anti-CD3 cross binding stimulation (**Figure 7B**). In the thymus, a significant effect of ERG1 blockage was evident upon stimulation (+), consisting in a significant reduction of Ca^2+^ intracellular levels (**Figure 7D**). This occurred in the subpopulations that responded to the stimulation: DP (p ≤ 0.01) and CD4SP (p ≤ 0.05) T cells. For comparison, we also evaluated the effects of Psora-4, a specific Kv1.3 blocker. Psora-4 was used for 2 h at [5 μM], a concentration shown to fully abrogate K_V_1.3 currents in mature CD4 T lymphocytes (53). No effects on intracellular Ca^2+^ concentrations upon T-cell stimulation was observed (**Figure 7D**).

### Effects of ERG1 inhibition on MAPK signal pathways

**Figure 8:**
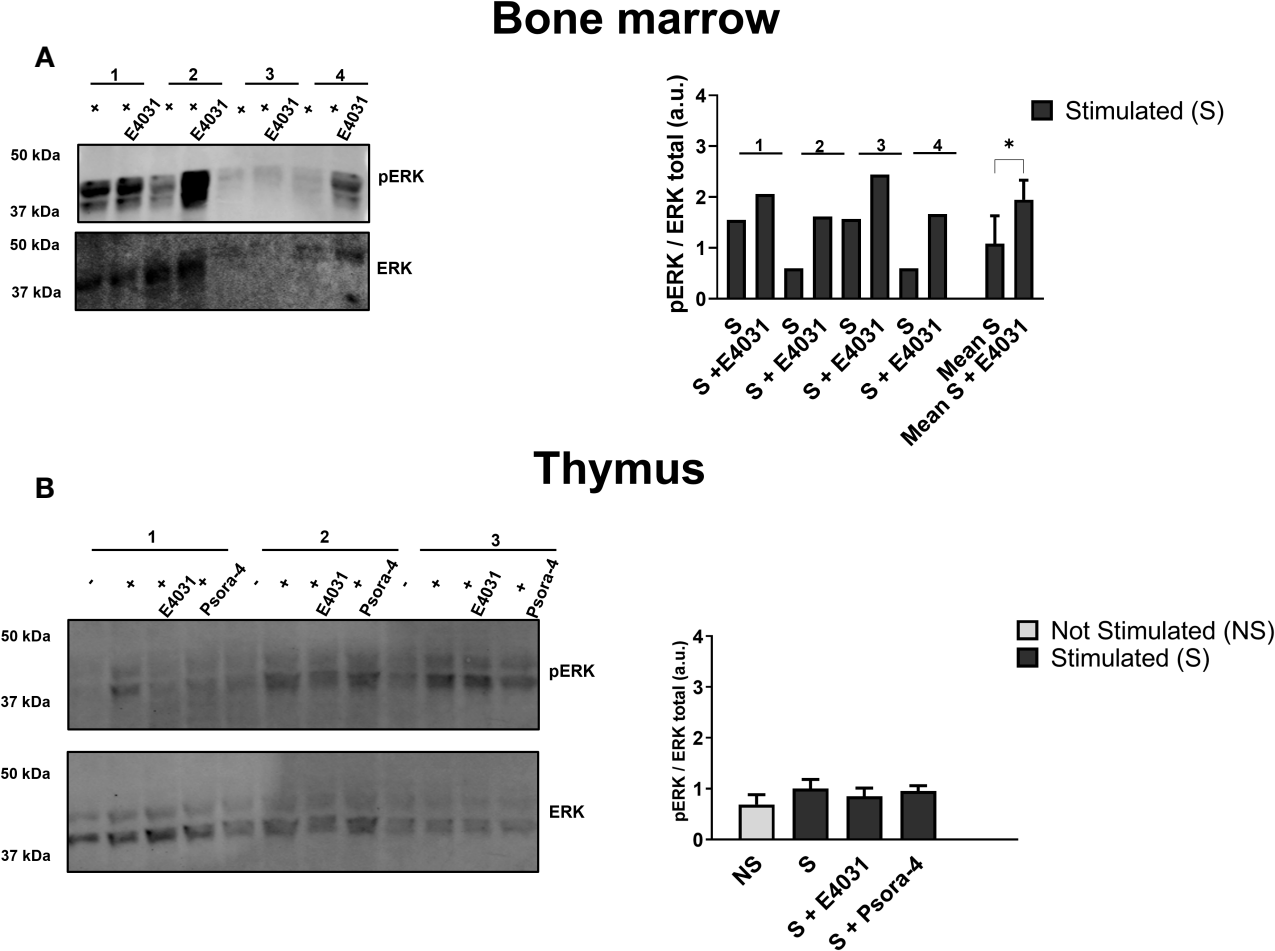
Effects of ERG1 inhibition on MAPK signal pathways Western blot analysis showing total ERK and ERK phosphorylation on residues Thr 202 and Tyr 204 on total lysates from BM **(A)** and thymus **(B)** of four C57BL/6 animals of 3 months stimulated and treated overnight with E4031 [30mM]. For each thymus (n = 4) are reported the unstimulated (−), stimulated (+), stimulated, and treated with E4031 and Psora-4 [5 mM] conditions. Histograms show the fold change in the phosphorylation calculated by WB quantification of pERK in each animal, normalized for the total ERK. Error bars represent standard deviation. Statistical analysis was performed by two-tailed T-test (0.01 < p ≤ 0.05 *).

We further evaluated the effect of E4031 and Psora-4 on other signaling pathways beyond Ca^2+^ influx. In particular, we focused on ERK phosphorylation, a pathway that is relevant during lymphocyte activation and development (11, 18) and is affected by E4031 ERG1 blockage in other cellular models (36, 54). ERK1/2 phosphorylation (pERK) on residues Thr 202 and Tyr 204 was evaluated by western blot (WB) on lysates from the whole BM and thymus in the presence or in the absence of E4031, incubated overnight, following BCR and CD3 stimulation. A significant increase in pERK after E4031 treatment was observed in the whole BM lysates from four C57BL/6 mice (**Figure 8A**). In the lysates from the thymus, where it was possible to include an unstimulated control and a stimulated sample treated overnight with Psora-4, an evident increase in pERK was observed following stimulation (i.e., CD3 crosslinking), whereas no effects of either E4031 or Psora-4 emerged (**Figure 8B**).

In summary, E4031 mERG1 K^+^ channel blockage produced a reduction of intracellular Ca^2+^ levels in the CD4^+^ and DP thymocyte precursors following stimulation, whereas it did not affect Ca^2+^ levels in the BM lymphoid subset, which, on the other hand, underwent an increase in ERK phosphorylation following mERG1 blockage.

## Discussion

In this work, we determined the expression profile of the K^+^ channel K_V_11.1, commonly known as ERG1, in different primary murine lymphoid populations. The expression of another voltage-dependent K^+^ channel, K_V_1.3, which is expressed in mature lymphocytes and whose relevance in lymphocyte activation is well known, was also analyzed. All the analyses were carried out in two different murine strains, C57BL/6 and SV129. This choice was driven by two considerations (1): the hematopoietic system may vary, in mice, depending on their strain (52), and (2) we had in mind to get insights from our results to be applied in studies related to different pathologies (either immune-mediated or oncologic). Worth noting is that different pathologies can be modeled in different mouse strains. Hence, it is not a surprise that we found some differences in the expression of both mERG1 and K_V_1.3 in the two mouse strains we analyzed. Such differences can be summarized as follows: much higher MFI values for both mERG1 and Kv1.3 were detected in C57BL/6 mice, compared with SV129 mice. Despite this, both mouse strains displayed a higher expression of mERG1 in the BM compared with the thymus. Furthermore, mERG1 turned out to be more expressed in DN T cells in the thymus of C57BL/6 mice, a fact which we did not detect in SV129 mice. Finally, mERG1 was expressed at higher levels in the B-cell compartment of SV129 mice compared with C57BL/6, but in both strains, the higher expression was observed in immature (large PreB/Small PreB) subpopulations. These differences must hence be taken into account when studying the role, and hopefully the relevance, of K^+^ channels in developing lymphocytes in the pathologic setting. Indeed, during the induction of EAE in the C67Bl6 strain, we observed that both mERG1 and Kv1.3 were also upregulated in the first phases of B-lymphocyte development and reduced in the latest IgM+ stage, suggesting a role of these channels during the onset of autoimmune encephalomyelitis. Based on these results, it is tempting to speculate that mERG1 dysregulation might be associated with the selection of self-antigens recognizing B-lymphocyte clones and to the onset of autoimmune diseases, representing an immunomodulator target similarly to Kv1.3 and KCa3.1 (16, 33).

Nevertheless, and interestingly, some differences in mERG1 and K_V_1.3 expression emerged from our data. In particular, a higher expression (higher MFI) of K_V_1.3 in the entire populations, either BM or thymus, emerged (compare **Figures 1C, D**, **3C, D**). Although this difference could be traced back to a different immunoreactivity of the two antibodies used to detect the two K^+^ channels in the present paper, interesting differences in the B subpopulations emerged, anyway. In particular, K_V_1.3 turned out to be more expressed in the IgM+ (C57BL/6) or large PreB (SV129) subpopulations, compared with mERG1. On the contrary, KV1.3 was expressed at low levels and did not show significant differences among the different subpopulations, in the T subsets. It is worth noting that, on the other hand, mERG1 was more expressed in the DN subpopulation in the thymus of C57BL/6 mice. Overall, these results suggest that mERG1 and K_V_1.3 could play partially overlapping, or even compensatory, functions during lymphocyte development. In particular, we hypothesize that the two voltage-gated K+ channels could collaborate in driving Ca^2+^ entry during lymphocyte selection processes.

We tested this possibility by analyzing the BCR and TCR Ca^2+^-mediated signaling mechanisms associated with lymphocyte selection checkpoints in C57BL/6 animals. To ensure that the variations in Ca^2+^ levels observed following stimulation were induced by Ca^2+^ flow across the CRAC channels on the plasma membrane and rule out a release from the intracellular store, BM and thymic cells were maintained in media supplemented with EGTA. This treatment completely abolished Ca^2+^ increase following stimulation, clearly indicating the primary role of Ca^2+^ influx from extracellular compartments while ruling out a major role of Ca^2+^ release from the internal store, as reported in literature (11, 55). It emerged that, in the thymus, the E4031 pharmacological blockage of mERG1 channels induced a reduction in intracellular Ca^2+^, in all the subsets that responded to the stimulation (DP and CD4+). On the contrary, no effect was detected in any BM subset. This suggests a role of the channel during the checkpoints of T lymphocyte maturation, where positive and negative selection and the lineage commission of cells expressing a correctly rearranged and non-autoreactive receptor take place. In this context, mERG1 could play a role in maintaining the electrochemical gradient responsible for driving Ca^2+^ entry during SOCE, similarly to Kv1.3. The latter is known to modulate the calcineurin-mediated NFAT dephosphorylation, regulating gene expression and crucial cellular responses like selection, differentiation, and activation of lymphoid progenitor cells (41). Therefore, it is tempting to speculate that the modulation of the activity of ERG1 might then impact a variety of intracellular processes that are central to cell-fate decisions during T-cell development. The perturbation of the Ca^2+^ signaling might lead to the proliferation of lymphocyte clones that would not normally be selected or that have arrested their development at an early stage of differentiation. This hypothesis is supported by a recent study although, in a different cellular context, showing a reduction of intracellular Ca^2+^ basal level following the pharmacological blockage of hERG1 currents by the specific blocker E4031 (56). Furthermore, our data indicating a role of ERG1 in maintaining the delicate balance between K^+^ and Ca^2+^ flow during T cell development, further stress the fact that an ERG1 dysregulation might then confer a survival advantage on proliferating autoreactive or tumorigenic clones similarly to Kv1.3 (15, 30, 31). This is in fact the case, i.e., in B-CLL, where both hERG1 and Kv1.3 overexpressions confer a survival advantage on proliferating lymphoid cancer cells (22, 34). Finally, other voltage-dependent Ca^2+^ channel-encoding genes such as CACNA1I, Cacnb2, and Cacna1f are expressed in human B and T lymphocytes (data available on https://www.proteinatlas.org). If the expression of the murine homologues of CACNA1I, Cacnb2, and Cacna1f was proven in murine lymphocyte precursors, further studies would be required to test an eventual contribution to the intracellular Ca^2+^ homeostasis, in conjunction with CRAC channels.

Furthermore, beyond Ca^2+^ signaling, when assessing the effect of mERG1 blockage on the MAP kinase signaling, a significant increase in ERK phosphorylation emerged in the BM. Future cytofluorimetric analysis of pERK in the different subsets will clarify if any specific developmental stage is particularly affected by E4031 blockage. Nevertheless, the observed increase in ERK phosphorylation in response to mERG1 inhibition suggests a role of the channel in reducing cell proliferation and might be associated with an enhanced differentiative process of the B-lymphocyte precursors, as suggested by the role of ERG1 during the development (45). In other words, mERG1 would participate in tuning the delicate balance between proliferation and differentiation, with relevant consequences both during lymphocyte development and the neoplastic transformation process. Furthermore, since abnormalities in the pERK signaling pathways have been linked to autoimmune disorders like systemic lupus erythematosus (SLE) (32), a role of ERG1 in these mechanisms could also be envisaged.

**Figure 9:**
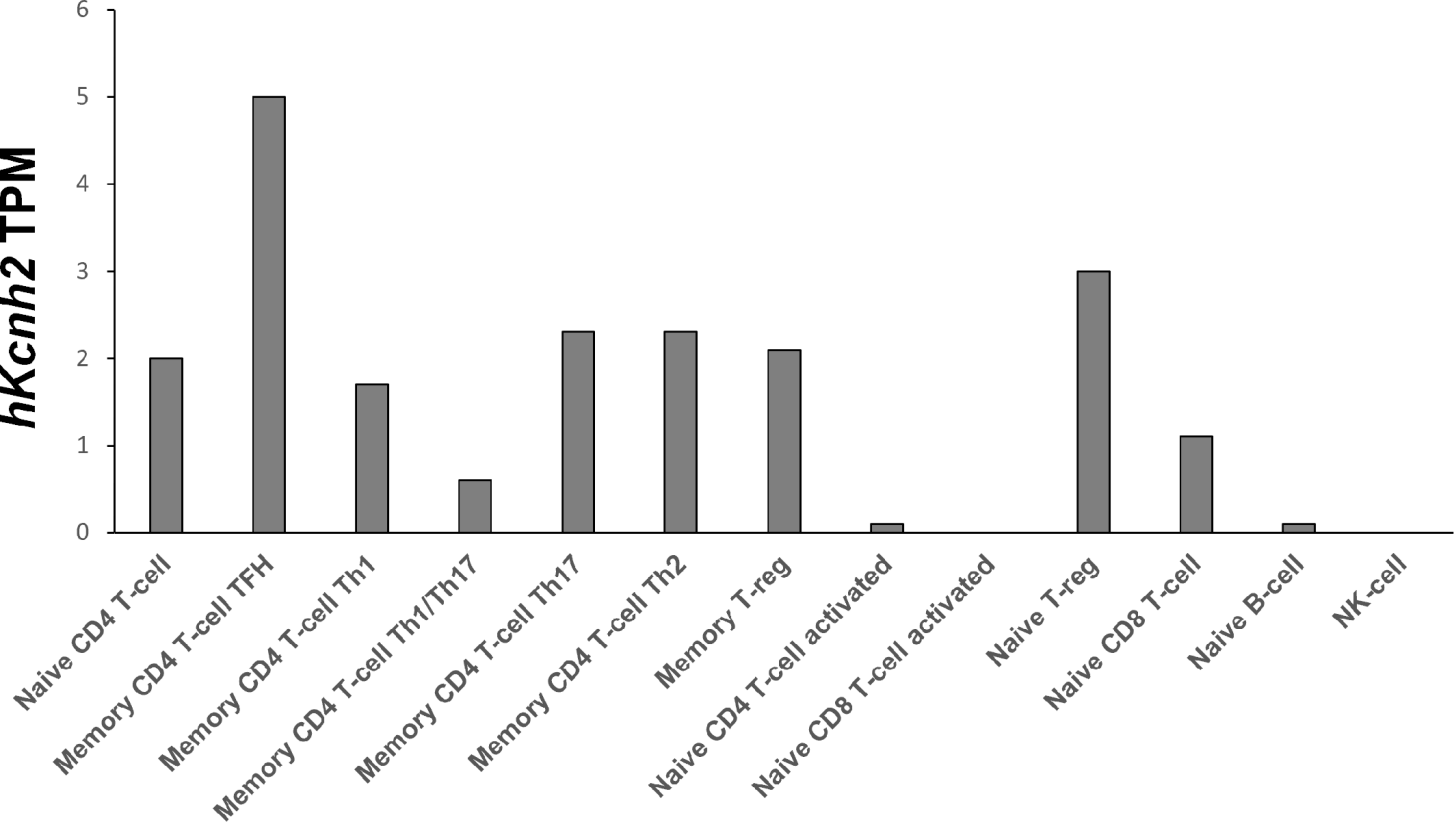
*hKCNH2* expression in different mature B and T lymphoid subsets Transcriptomic analysis based on RNA-seq data of 13 types of human immune cells from 91 donors, deposited on the DICE database (https://dice-database.org), *showing hKCNH2* transcripts per million (TPM*)*.

To our knowledge, this is the first detailed study on the expression of Kv1.3 and ERG1 conducted on specific lymphocytes’ precursor subsets. In particular, we here identified for the first time the expression profile of mERG1 K^+^ channels in different murine lymphocyte subtypes. Our results are partially corroborated by publicly available RNA-seq data of 13 types of human immune cells from 91 donors deposited on the DICE database (database of immune cell expression, expression, quantitative trait loci, and epigenomics) by Schmiedel and colleagues (57). Despite not including lymphocyte precursor subsets, the transcriptomic analysis identifies hERG1 expression in different mature B and T human lymphoid subsets (**Figure 9**). Interestingly, one of the subsets highly expressing the hERG1 encoding gene *KCNH2* is the Treg compartment, further stressing the possibility that this channel would exert a role in the onset of autoimmune diseases, hence representing a novel biomarker.

## Data availability statement

The raw data supporting the conclusions of this article will be made available by the authors, without undue reservation.

## Ethics statement

The animal study was approved by The study’s protocol was approved by the Italian Ministry of Health with the authorization number 721/2019. The study was conducted in accordance with the local legislation and institutional requirements.

## Author contributions

CS and AA: designed research. CS, MS, and TL: performed research. CS and MS: analyzed data. GA and GC provided the organs from EAE mouse model. CD identified the binding region of the anti-ERG1 mAb. CS and AA: edited and wrote the paper. All authors contributed to the article and approved the submitted version.
