# Supplementary material for: Expression of the ether-a-gò-gò-related gene 1 channel during B and T lymphocyte development: role in BCR and TCR signaling
Source: Front Immunol. 2023 Sep 8;14:1111471. doi: 10.3389/fimmu.2023.1111471 (PMC10515723; doi:10.3389/fimmu.2023.1111471)
Supplement: Supplementary file 1 [file DataSheet_1.pdf]

# *Supplementary Material*

# Expression of the ether-a-gò-gò-related gene 1 channel during B and T lymphocyte development: role in BCR and TCR Ca2+ signalling

**Cesare Sala*, Martina Staderini, Tiziano Lottini, Claudia Duranti, Gabriele Angelini, Gabriela Constantin, Annarosa Arcangeli**

*** Correspondence:** Cesare Sala: cesare.sala@unifi.it

## 1.1 Supplementary Figures

### Supplementary Figure 3: Sequence alignments between the human and mouse proteins of hERG1

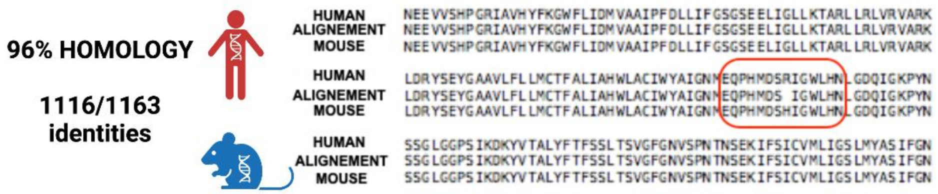


Aminoacidic sequence alignment showing the homology between the human and mouse ERG1 protein. The first line shows human aa sequence, the second line shows the overlapping between the first and third line, the latter shows the mouse aa sequence. In the red square is highlighted the epitope recognized by the monoclonal anti-ERG1 antibody used in the FC experiment.

**Supplementary Figure 2: Lymphocytes' precursors gating strategy.**

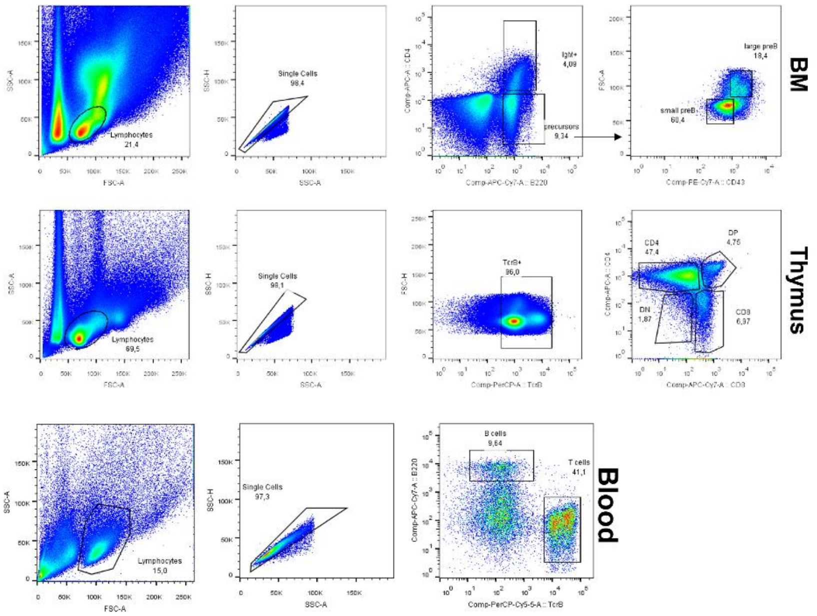


Representative plots showing the gating strategy used to identify the different lymphocytes subsets purified from thymus, BM and blood of a 3 months old male SV129 mouse. BM B cell precursors that have not yet expressed IgM (B220+ IgM-), have been subdivided into the large pre-B (Hardy's fraction C') and small pre-B cell (Fraction D) subsets on the base of their dimension and of the expression of the CD43 marker, and immature B cells that express IgM (B220 + IgM +, Fraction D). Thymocytes have been first gated on TCRβ+ (intermediate and high expression level) and then further subdivide

into double negative (DN), double positive (DP), and CD8 and CD4 SP subsets, according to the expression of the CD4 and CD8 coreceptors markers.
Mature cells from blood have been subdivided into B cells and T cells based on the respectively expression of B220 and TCRβ.

**Supplementary Figure 3: ERG1 and Kv1.3 expression in different lymphoid populations of older mice.**

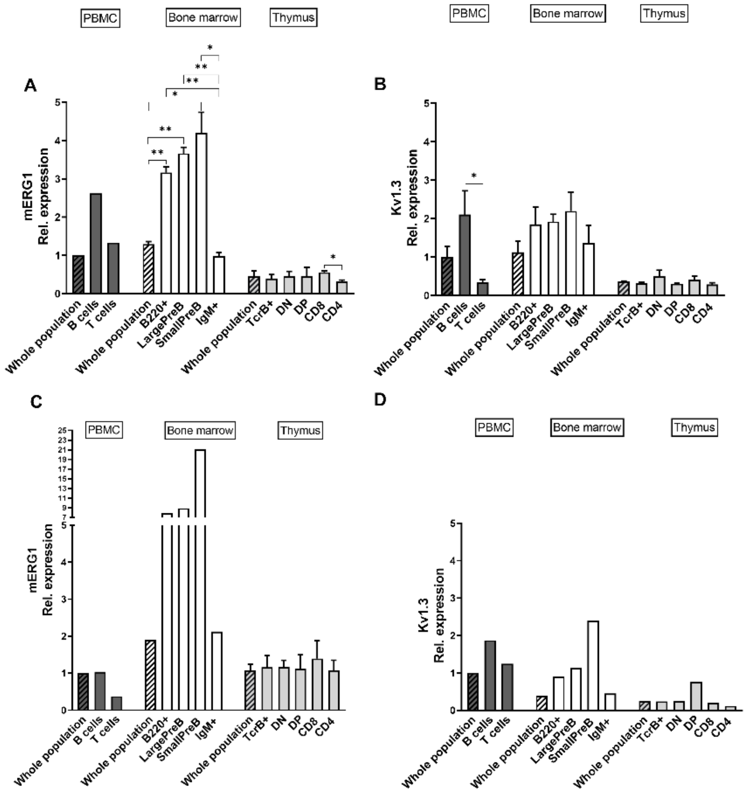


Representative histograms showing anti-ERG1 (AF488) and anti-Kv1.3 (AF488) fluorescence analysed by flow cytometry in the different lymphoid precursors' subpopulations purified from different lymphoid organs of SV129 mice from different age.
Histograms representing ERG1 expression levels in different lymphoid organs (bars marked with line pattern) and subsets (bars with no pattern, gated as described in Supplementary Figure 1) of 7 months old **(**n.=2 **A)** and 14 months old **(**n.=1 **C)** animals of both sexes, normalized on the ERG1 MFI of the PBMC.
Histograms representing Kv1.3 expression levels in different lymphoid organs (bars marked with line pattern) and subsets (bars with no pattern, gated as described in Supplementary Figure 2) of 7 months

old **(**n.=2 **B)** and 14 moths old **(**n.=1 **D)** animals of both sexes, normalized on the Kv1.3-AF488 MFI of the PBMC.
Histograms represent the mean of the fluorescence analysed in the different animals, and error bars indicate the standard deviation.
Statistical analysis was performed by two-tailed T-test ($0.01 < p \leq 0.05$ *, $0.001 < p \leq 0.01$ **, $p \leq 0.001$ ***).

**Supplementary Table 1**

| n. | birth | immunized | sex | sacrificed | EAE score |
|---|---|---|---|---|---|
| 1 | 02/11/2022 | 09/01/2023 | m | 26/01/2023 | 2 (Peak) |
| 2 | 02/11/2022 | 09/01/2023 | m | 26/01/2023 | 2,5 (Peak) |
| 3 | 02/11/2022 | 09/01/2023 | m | 26/01/2023 | 2 (Peak) |

Table reports the data of the 3 months old C57/BL6 murine model of induced EAE.
